# Supplementary material for: Interaction Among Influenza Viruses A/H1N1, A/H3N2, and B in Japan
Source: Int J Environ Res Public Health. 2019 Oct 29;16(21):4179. doi: 10.3390/ijerph16214179 (PMC6862093; doi:10.3390/ijerph16214179)
Supplement: Supplementary file 1 [file ijerph-16-04179-s001.pdf]

Supplementary materials

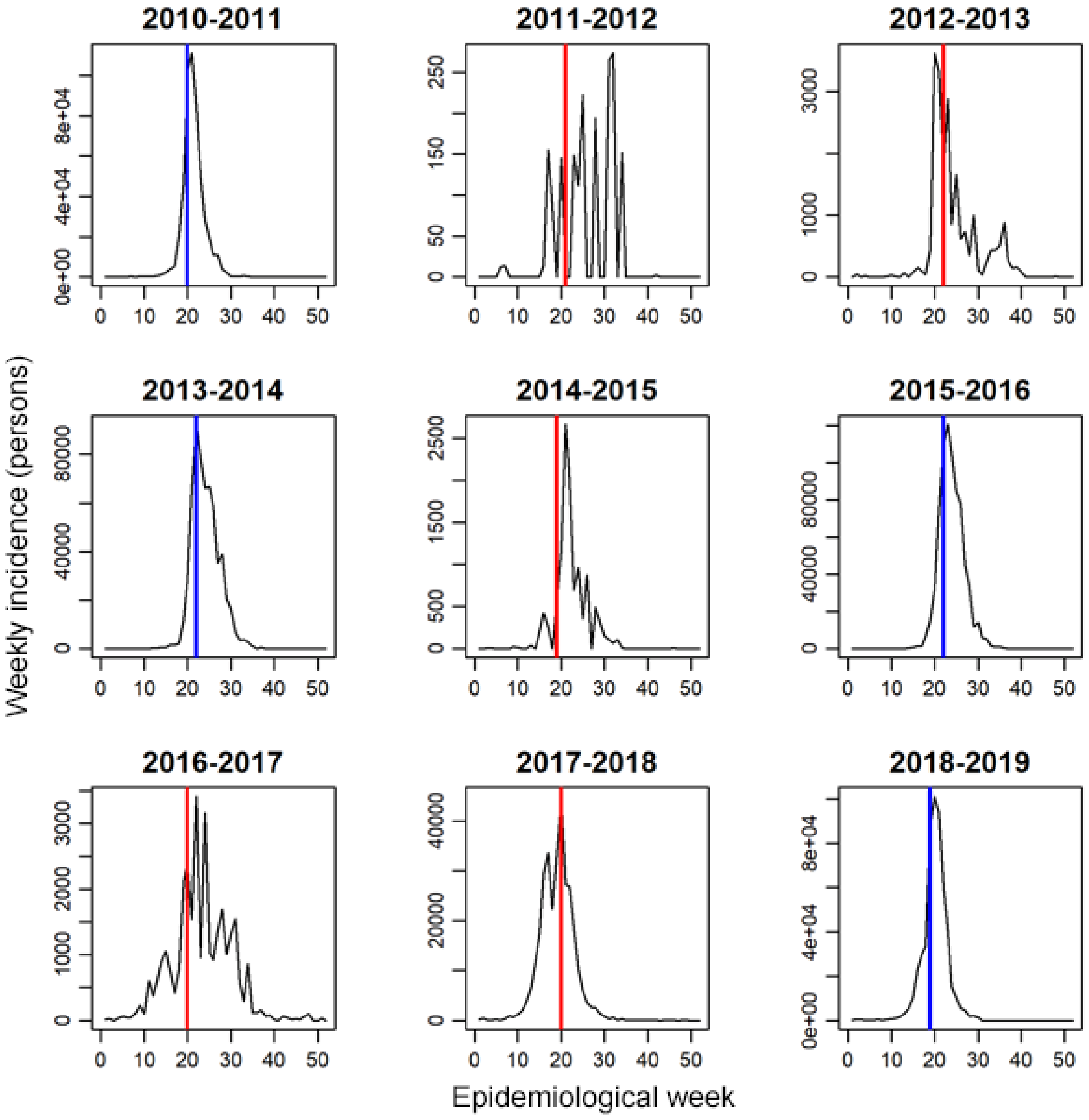

Figure S1. Temporal distribution of the weekly incidence proxies for influenza A/H1N1 from 2010 to 2019. Epidemiological week 1 corresponds to calendar week 36. Blue lines indicate the prediction timing of the epidemic size for the season, corresponding to the time at which the sum of the incidence of influenza A/H1N1 in the past 5 weeks exceeds the statistically chosen threshold h=250,000 cases. Red lines indicate the prediction timing corresponding to the time at which the complementary cumulative incidence from week 1 exceeds the chosen threshold h_c=570,000 cases.

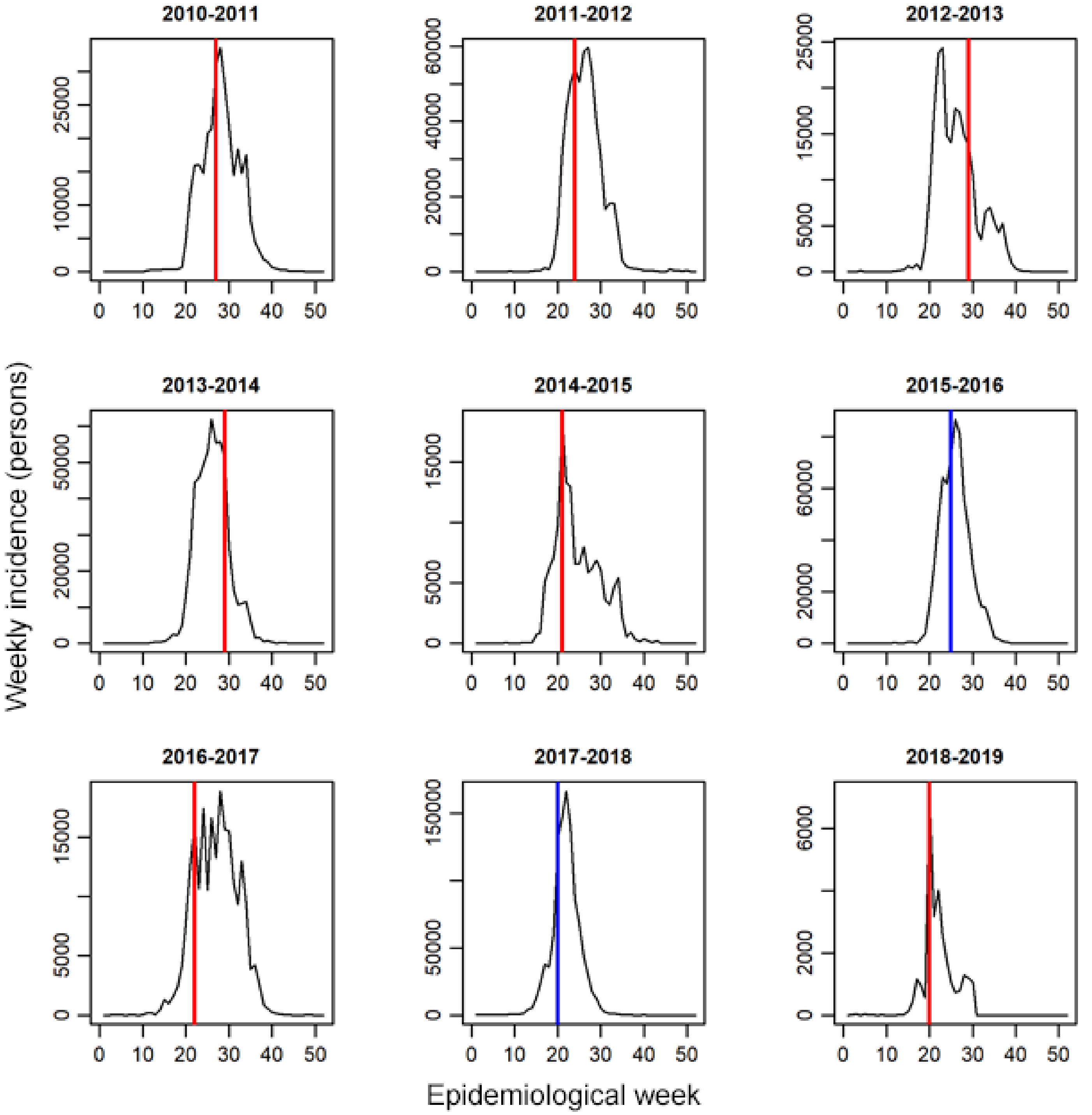

Figure S2. Temporal distribution of the weekly incidence proxies for influenza B from 2010 to 2019. Epidemiological week 1 corresponds to calendar week 36. Blue lines indicate the prediction timing of the epidemic size for the season, corresponding to the time at which the sum of the incidence of influenza B in the past 5 weeks exceeds the statistically chosen threshold h=310,000 cases. Red lines indicate the prediction timing corresponding to the time at which the complementary cumulative incidence from week 1 exceeds the chosen threshold h_c=810,000 cases.

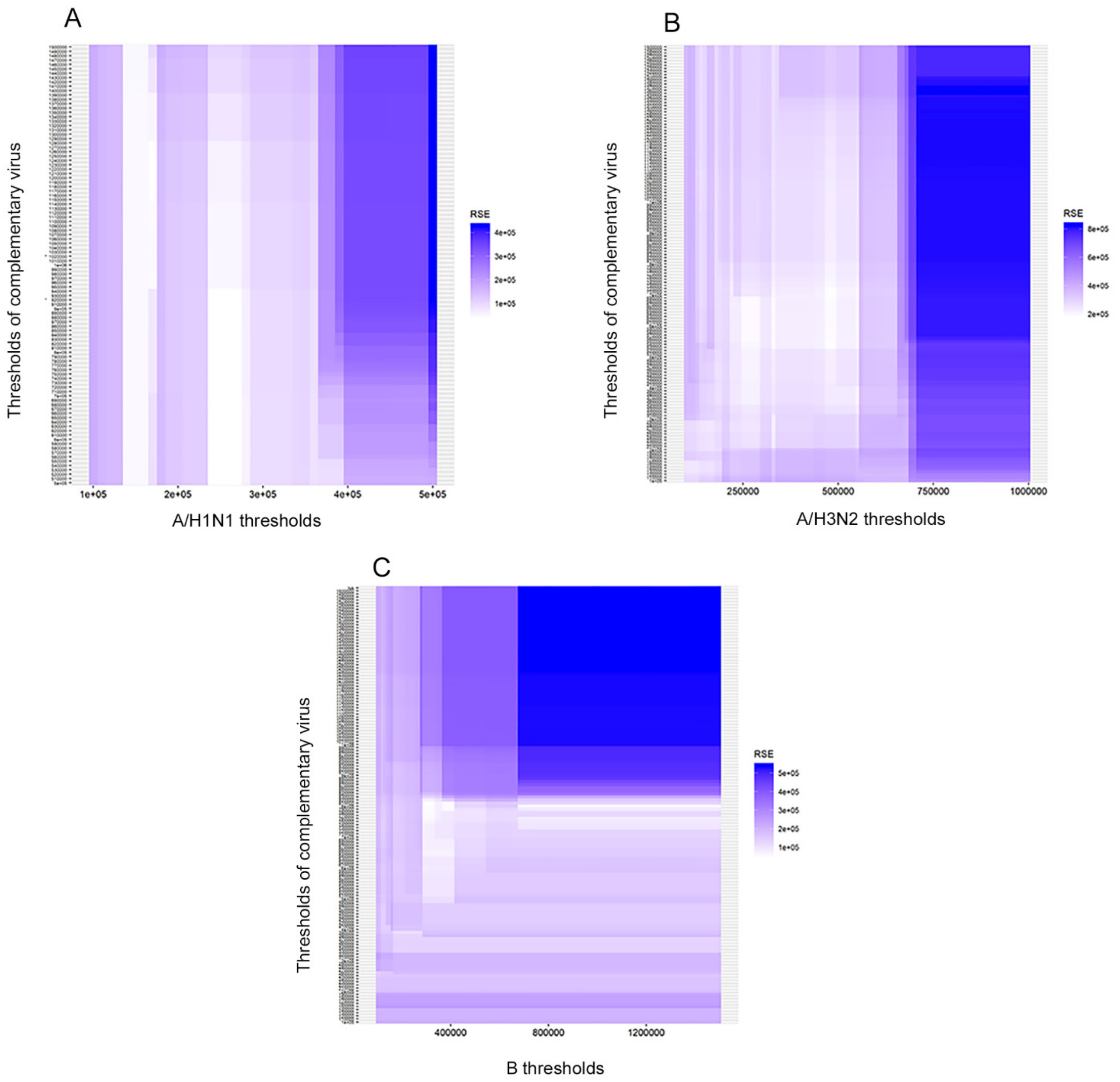

Figure S3. Residual standard error for various combinations for thresholds *h* and *h*$_c$.

(A) Influenza A/H1N1; (B) Influenza A/H3N2; (C) Influenza B.

Table S1. Summary of the prediction timing of week s and the epidemic peak weeks of influenza A/H1N1, A/H3N2, and B

	A/H1N1		A/H3N2		B	
	week s	peak week	week s	Peak week	week s	Peak week
2010	20	21	28	28	27	28
2011	21	32	22	22	24	27
2012	22	20	22	21	29	23
2013	22	22	25	22	29	26
2014	19	21	19	21	21	21
2015	22	23	24	25	25	26
2016	20	22	21	21	22	28
2017	20	20	21	21	20	22
2018	19	20	20	21	20	20
